# Acute-Onset Postoperative Herpetic Endophthalmitis: A Case Report

**DOI:** 10.7759/cureus.40875

**Published:** 2023-06-23

**Authors:** Vinita Gupta, Umesh Yadav, Saurabh Luthra, Anurag Singla

**Affiliations:** 1 Ophthalmology, All India Institute of Medical Sciences, Rishikesh, Rishikesh, IND; 2 Ophthalmology, Drishti Eye Institute, Dehradun, IND

**Keywords:** post cataract surgery endophthalmitis, complicated cataract surgery, polymerase chain reaction, herpetic endophthalmitis, acute post operative endophthalmitis

## Abstract

Herpes simplex virus uveitis without corneal reactivation is more frequent than previously thought. Although herpes simplex virus has been implicated as a cause of postoperative uveitis and endophthalmitis, it has not been reported as a cause of acute postoperative endophthalmitis within the early postoperative period, specifically within one week following cataract extraction. A 55-year-old man with vascularized irregular central disc-shaped stromal corneal opacity with complicated cataracts underwent cataract surgery. Intraoperatively, there was posterior capsular rent, requiring anterior vitrectomy. On postoperative day three, the patient had an increase in inflammation in the anterior chamber (grade 4+) with marked vitreous haze (grade 4). Vitreous taps were negative for bacteria and fungi, and despite intravitreal injections of vancomycin and ceftazidime, the patient had worsening of inflammation with increasing exudates and the appearance of the fibrinous membrane in the anterior chamber. Polymerase chain reaction (PCR) of aqueous and vitreous samples at this point of time yielded positive serology for herpes viral DNA, and the patient was started on oral valacyclovir. The ocular inflammation resolved soon after switching to oral valacyclovir. Typical acute postoperative endophthalmitis starts two to seven days after surgery, and the most common isolate in vitreous biopsies is coagulase-negative staphylococci. We report a rare case of acute-onset herpetic endophthalmitis presenting within 72 hours following cataract surgery for a complicated cataract in a patient with a history of pre-existing healed viral keratitis. Our case highlights that a suspicion of viral endophthalmitis should be kept in mind as a cause of acute-onset post-cataract surgery endophthalmitis, especially in cases of surgery that fail to yield a positive result on Gram’s stain, culture or PCR for bacteria and fungi.

## Introduction

Acute infectious endophthalmitis after cataract extraction is a rare but potentially blinding complication. The overall incidence of post-cataract surgery endophthalmitis has declined over the past centuries. The incidence of endophthalmitis after cataract surgery was approximately 5% to 10% in the late 1800s and early 1900s, 1.5% to 2% during the 1930s, 0.5% to 0.7% in the mid-1900s, and 0.06% to 0.09% in the early 1990s, according to nationwide patient registries of United States [1-5]. In more recent studies from developed countries, reported incidence rates of post-cataract surgery acute endophthalmitis vary between 0.04% and 0.12% [6-8]. Advancements in surgical materials, improvements in microsurgical and aseptic techniques, and the use of prophylactic broad-spectrum antibiotics explain this favorable trend. The incidence of acute endophthalmitis after cataract extraction in Asian populations is also consistent with rates reported elsewhere. Sun et al., looking at the incidence trends of post-cataract surgery endophthalmitis from 2008 to 2019, found the incidence rate to be 0.076% [9]. In another study of a multi-ethnic Asian resident population, the average annual incidence of acute endophthalmitis has been reported to be 0.076% [10].

The major organisms for acute postoperative endophthalmitis as reported are coagulase-negative staphylococci (70%), *Staphylococci aureus* (10%), Streptococci (9%), other Gram-positive cocci, including enterococci, mixed bacteria (5%), and Gram-negative bacilli (6%) [11,12]. In tropical countries like India, fungi may cause up to 10-15% of cases [13]. *Propionibacterium acnes* has been reported to cause chronic post-cataract endophthalmitis [11]. In developing countries, there is a notable high prevalence of bacterial and fungal keratitis, as well as endophthalmitis, following cataract surgery. Consequently, the focus of research and study has been primarily directed toward these prevalent infections, leading to a relative lack of emphasis on investigating cases of keratitis and endophthalmitis specifically associated with the herpes simplex virus (HSV). In developed countries as well, although herpes simplex virus (HSV) is widely recognized as the leading infectious cause of blindness, it has not historically been linked to acute postoperative endophthalmitis occurring within the first week following cataract surgery [14]. We describe here a rare case of acute-onset endophthalmitis occurring within 72 hours following cataract surgery as a result of the herpes virus. 

## Case presentation

A 55-year-old male presented to us with gradual, progressive, painless decrease in vision in the right eye (RE) for one year. Fifteen years ago, the patient had experienced a previous episode of painful decreased vision in the RE, accompanied by redness, and this was followed by the development of a corneal scar. There was no history of any ocular complaints in the left eye (LE). He had undergone an uneventful phacoemulsification with posterior chamber intraocular lens implantation in the LE five years ago. There was no history of trauma, immunosuppression, or travel to an endemic area. There was no history of oral herpes.

**Figure 1:**
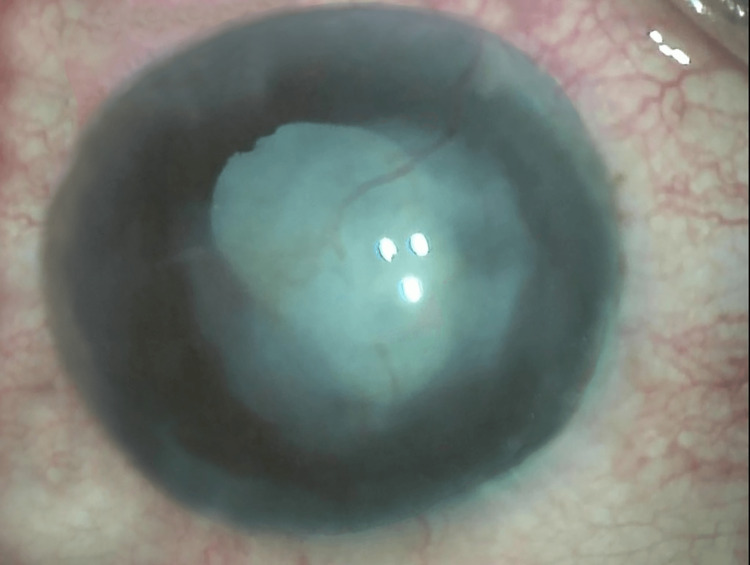
Biomicroscopic slit lamp photograph of the right eye's anterior segment (diffuse illumination) at presentation showing vascularized irregular central disc-shaped stromal corneal opacity of mild to moderate density with total complicated cataract.

On examination, the patient had a best corrected visual acuity (BCVA) of hand movements close to the face in the RE and 6/9 in the LE. The intraocular pressure was normal in both eyes. There was a complete cataract in the RE with a severity grading of 5 based on the nuclear color and opalescence as per the Lens Opacification Classification System III. Furthermore, there were significant posterior synechiae and a pupil that exhibited poor dilation. In addition, a vascularized irregular central disc-shaped stromal corneal opacity of mild to moderate density was present (Figure 1). The corneal opacity was associated with reduced corneal sensations, indicating a healed disciform keratitis. There was no view of the posterior segment. Ultrasound (USG) B-scan revealed an anechoic vitreous cavity with a retina that appeared attached. The left eye had pseudophakic status and a normal fundus examination. With the clinical diagnosis of complicated cataract with healed herpetic keratouveitis in the right eye, the patient was scheduled for right eye cataract surgery with prophylactic antiviral and corticosteroid therapy. Oral acyclovir 400 mg was initiated twice a day, one week before the surgery for the prevention of reactivation of herpetic stromal keratitis. Corticosteroid prophylaxis with oral prednisolone 40 mg once a day was also started along with topical prednisolone acetate 1% eye drops four times a day and nepafenac 0.1% eye drops twice a day in the RE three days prior to surgery. During the surgery, a posterior capsular rent and zonular dialysis were noted after the removal of the cataractous lens. A limited anterior vitrectomy was done, and the patient was left aphakic.

**Figure 2:**
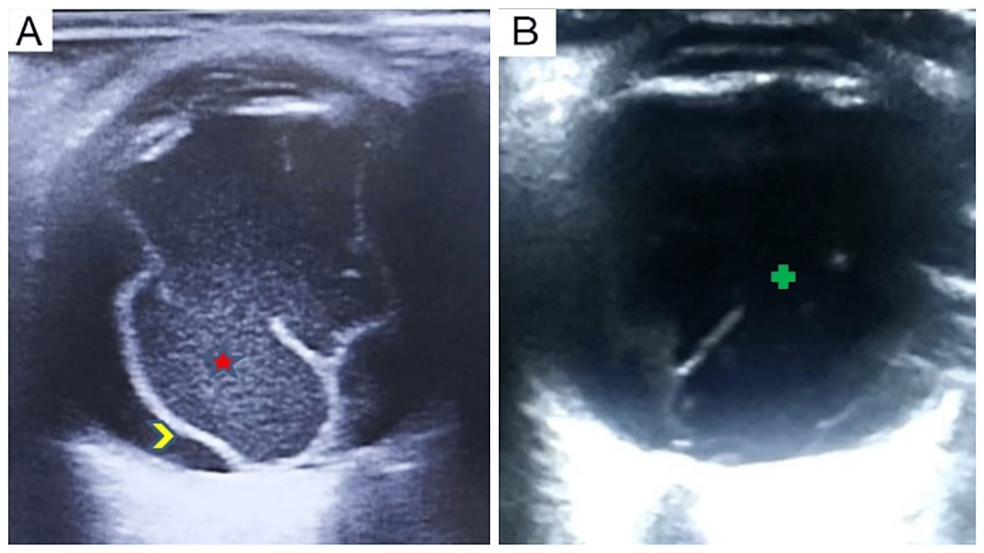
Ultrasound B-scan images of the posterior segment of the right eye. (A) On the third postoperative day showing multiple low to moderate reflectivity echoes in the vitreous cavity (red star) with an undulating moderately reflective membrane (yellow arrow-head) with good after movements suggestive of severe vitreous inflammation, posterior vitreous detachment and an attached retina. (B) At two weeks post-antiviral treatment showing significant resolution of vitreous echoes (green plus).

On the first day after the surgery, the patient's BCVA was 6/60 with 2+ anterior chamber inflammation and a few Descemet membrane folds. There was a good fundal glow with no vitreous inflammation, a normal appearance of the optic disc, unremarkable retinal vessels, a normal macula, and an attached retina. Oral acyclovir 400 mg twice a day along with topical moxifloxacin 0.5% eye drops four times a day and nepafenac 0.1% eye drops three times a day were continued. The frequency of topical prednisolone acetate 1% instillation was increased to every three hours, and homatropine 2% was added three times a day. However, on the third postoperative day, there was a progressive increase in anterior chamber inflammation. This was evident by circumcorneal congestion, the presence of 4+ cells in the anterior chamber, and the observation of a 1 mm hypopyon. Additionally, there was a loss of fundal glow. Notably, there were no accompanying symptoms such as pain, excessive tearing, or sensitivity to light. There was no swelling of the eyelids or conjunctival chemosis. USG B-scan (Figure 2A) revealed the presence of multiple low to moderate reflectivity echoes in the vitreous cavity suggesting the presence of severe vitreous inflammation.

**Figure 3:**
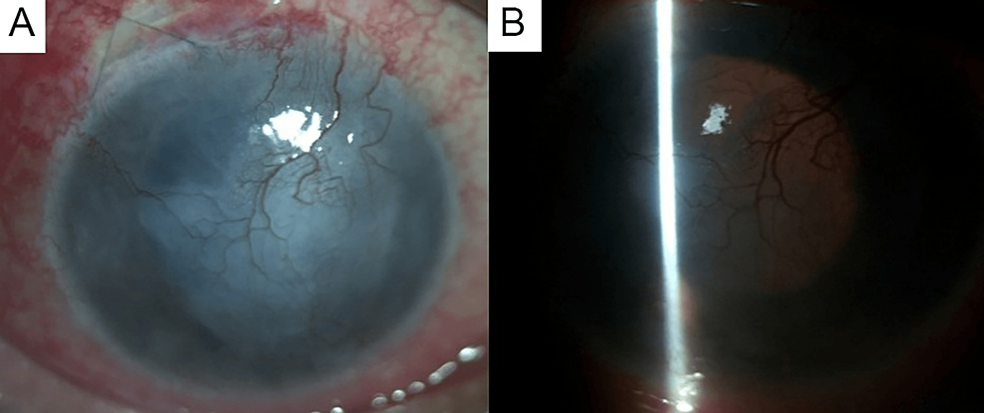
Biomicroscopic slit lamp photograph of the right eye's anterior segment. (A) Diffuse illumination at two weeks post-antiviral treatment showing resolving circumcorneal congestion with vascularization of the central disc-shaped corneal scar and resolving anterior chamber inflammation. (B) Retro illumination at five weeks post-initiation of antiviral treatment showing a vascularized macular corneal scar with a good fundal glow.

Additionally, an undulating membrane with moderately reflective echoes was observed with good after movements, consistent with posterior vitreous detachment. The retina was found to be attached. With the presumptive diagnosis of severe postoperative sterile inflammation, the frequency of topical prednisolone acetate eye drops instillation was increased to one-hourly intervals, and oral prednisolone was increased to 80 mg per day along with subconjunctival injections of gentamicin 40 mg/0.5 ml and dexamethasone 2 mg/0.5 ml. After 24 hours, with no improvement in the anterior and posterior segment inflammation, aqueous and vitreous taps were taken and sent for microbiological analysis (Gram’s stain, KOH mount and culture on blood agar, chocolate agar, Sabouraud’s dextrose agar, thioglycolate broth, and brain heart infusion broth). Intravitreal injections of vancomycin (1 mg/0.1 ml), ceftazidime (2.25 mg/0.1 ml), and dexamethasone (0.4 mg/0.1 ml) were administered. Microbiological analysis yielded negative results for bacterial growth in both smear and culture. Additionally, there were no fungal elements detected in the KOH preparation or through polymerase chain reaction (PCR). Over the subsequent 48 hours, there was a progression of exudates in the anterior chamber and the emergence of a fibrinous membrane in the pupillary area. With signs of worsening inflammation in the anterior segment but no worsening of inflammation in the posterior segment on the USG B-scan, viral endophthalmitis (secondary to reactivation of herpes simplex) was suspected, and repeat aqueous and vitreous taps were taken and sent for a Tzanck smear and PCR for herpes DNA. Pathological examination of the ocular fluids did not reveal any multinucleated giant cells; however, PCR testing of the fluids was positive for herpes simplex viral DNA. Oral valacyclovir, 1000 mg every eight hours, was added to the patient's treatment regimen, and a positive response was noted with decreasing inflammation over the next two weeks (Figures 2B, 3A). There was a complete resolution of inflammation in the subsequent three weeks, resulting in a best-corrected visual acuity of counting fingers at 5 meters. There was a good fundal glow (Figure 3B) and a normal-looking retina.

## Discussion

The incidence of acute postoperative endophthalmitis after cataract surgery has steadily declined from 2% in the first half of the 20th century to current levels ranging from 0.03% to 0.2% [15]. Several risk factors like the patient’s demographics-old age, rural residence, immunosuppressive condition, presence of blepharitis and ectropion determine the severity and outcome of postoperative endophthalmitis [16]. Intraoperative risk factors reported are intracapsular cataract surgery, vitreous loss, and anterior vitrectomy. Also, polymethyl methacrylate and silicone lenses have a fourfold increased risk as compared to acrylic lenses. Furthermore, surgical complications like posterior capsular rent increase the risk by two to three folds [17].

Ocular viral infections can appear as isolated ocular manifestations or as part of systemic infection with Varicella Zoster Virus (VZV), Herpes Simplex Virus (HSV-1), Epstein Barr Virus (EBV), and Cytomegalovirus (CMV). HSV is the most common infective cause of blindness in developed countries, with a reported incidence of 5.9-20.7 per 100,000 person each year [18]. In a study by Shah et al., among all cases of herpes simplex keratitis, 17.5% presented with epithelial keratitis, 51.6% with stromal keratitis (stromal keratitis with ulceration), 7.2% with stromal keratitis without ulceration, 21.8% with endotheliitis (15.9% as disciform keratitis, 5.9% as keratouveitis), and 5% with neurotrophic keratitis [19]. Herpes zoster ophthalmicus presents as keratitis in 31.4%, keratouveitis in 31.4%, isolated anterior uveitis in 29.4%, and optic neuritis in 1.9%. Corneal involvement presents as stromal keratitis in 15.6%, epithelial keratitis in 9.8%, and concomitant superficial and stromal keratitis in 5.8% [20]. Viral infections also represent a significant cause of posterior segment endogenous viral endophthalmitis (EVE) due to their systemic spread, and viruses are more likely than other organisms to spread via a neuronal pathway. Several viruses have been implicated in the development of EVE, most commonly VZV, HSV I and II, CMV, and EBV. Multiple factors influence the outcome of viral reactivation like human leucocyte antigen, host immune response, and virulence of the strain [21]. EVE responds well to systemic antivirals and steroids with good resolution of symptoms.

Polymerase chain reaction (PCR) is the main laboratory test that is effective in establishing the diagnosis of ocular viral infections due to HSV, VZV, or CMV. PCR was found to be 91.3% sensitive and 98.8% specific for the diagnosis of HSV-1, HSV-2, VZV, EBV, and CMV from aqueous and vitreous samples. Viral serology of the vitreous is effective in confirming the pathogen involved in 80-90% of cases [22].

Recurrence rates of ocular HSV after an initial episode have been estimated as 10% at one year, 23% at two years, 36% at five years, and over 60% at 20 years [23]. Many factors have been attributed to HSV reactivation, including ocular surgery, epithelial debridement, stress, trauma, ultraviolet radiation, heat stress, and various medications, particularly corticosteroids that trigger viral replication. Cataract surgery is one of the various stimulants that can reactivate the virus and cause infection even without a previous history of herpetic eye disease. The combination of surgical trauma along with topical corticosteroid treatment commonly prescribed after surgery may trigger reactivation. Periodic reactivations in the cornea (HSV keratitis) after cataract surgery are known [24-26]. Recurrence of herpes simplex uveitis after phacoemulsification surgery has also been reported [27].

Excessive postoperative inflammation may occur, especially after cataract surgery in patients with uveitic eyes. The postoperative course can be complicated by the formation of fibrinous membranes in the anterior chamber, especially in patients who had excessive manipulation intraoperatively, as in our case. Inflammation involving both the anterior and posterior segments in our patient was initially thought to be sterile uveitis and was treated by topical, depot, and systemic steroids. However, because of worsening inflammation over the subsequent two days, infective endophthalmitis was considered. There are a limited number of case reports documenting HSV as an etiology for postoperative endophthalmitis. Al-Ani et al. found recurrence of keratitis and uveitis postoperatively in 17 of 37 patients (45.9%) with HSV-related keratitis and/or anterior uveitis who underwent subsequent cataract surgery [27]. Half of these recurrences occurred in the first year. They also found one case of postoperative endophthalmitis in their retrospective series. Yang et al. reported a case of herpetic endophthalmitis in a 65-year-old man after uneventful phacoemulsification [28]. However, their case was presented in the fourth week after the surgery and was diagnosed as HSV endophthalmitis after serological testing of the capsular bag. Hu et al. found significant inflammation in the anterior chamber of the right eye along with moderately dense vitreous opacity in their immunocompetent older female patient after phacoemulsification cataract surgery [29]. In their patient, vitreous opacification was observed starting on the 26th day and fibrinous exudation in the anterior chamber on the 34th day following the cataract surgery. The diagnosis of herpetic uveitis was made with an aqueous humor examination showing positive results for herpes simplex virus DNA by PCR. Our patient developed endophthalmitis within 72 hours following cataract surgery. This could be explained because our patient had pre-existent healed disciform keratouveitis (presumably post-HSV) and complicated cataract surgery (posterior capsular rent and anterior vitrectomy). While patients of Yang et al. and Hu et al. had uneventful phacoemulsification surgeries, and there was no history of prior ocular viral disease. Al-Ani et al., in their retrospective series of 37 eyes, also found one case of postoperative endophthalmitis occurring on day three of cataract surgery. However, the vitreous sample from their case did not grow any organisms and was negative for viruses and toxoplasmosis on PCR testing. In our case, both aqueous and vitreous samples were positive for herpes simplex viral DNA on PCR testing.

Various case series suggest that prophylactic antiviral therapy with acyclovir or valacyclovir should be initiated before surgery when preparing the eye for cataract removal in patients with herpetic uveitis, but the validity of antiviral prophylaxis has not been extensively studied [24-25,27]. Furthermore, there are no specific guidelines on the timing of drug initiation, dosage, and duration of treatment. Sykakis et al. [26], in a questionnaire-based survey to ascertain the clinician's management of a patient with known HSV disease having cataract surgery, found that 58.8% of surgeons would start the patient on oral antiviral treatment. The perioperative antiviral therapy prescribed would be oral acyclovir 400 mg twice daily. About 67.5% of the respondent surgeons would start preoperatively, and 69.7 % would continue the same regimen postoperatively for 30 days. Recently, Rodriguez-Garcia et al. [30] have recommended that systemic antiviral therapy with acyclovir or valacyclovir should be administered at least one week before surgery to prevent recurrent viral infection in cases of HSV-related keratitis and/or anterior uveitis in patients undergoing subsequent cataract surgery. They have also recommended that antivirals should be changed to therapeutic dose for seven to 14 days postoperatively and then reduced to prophylactic levels (acyclovir, 600-800 mg/day and valacyclovir, 500-1000 mg/day) for several weeks to months before stopping them. In our case, we also started the patient on corticosteroid and antiviral prophylaxis one week before the surgery. We started with oral acyclovir 400 mg twice a day and continued with the same in the immediate postoperative period. However, when our patient had postoperative endophthalmitis, we switched to the full therapeutic dose of valacyclovir, one gram three times a day, which was continued for eight weeks.

Our case report focuses on a patient with HSV-related keratouveitis who developed postoperative endophthalmitis on the third day following complicated cataract surgery. The diagnosis of herpetic etiology was confirmed through vitreous PCR testing. Our case emphasizes the importance of considering viral endophthalmitis as a potential cause of acute-onset post-cataract surgery endophthalmitis within the first week after surgery, particularly in instances where Gram's stain, culture, or PCR testing for bacteria and fungi yield negative results.

## Conclusions

HSV uveitis without corneal reactivation is more frequent than previously thought. Cataract surgery is one of the various stimulants that can reactivate the virus and cause infection. There is a significant risk for recurrent inflammation in the first year postoperatively after cataract surgery. Besides uveitis, HSV may also cause acute-onset postoperative endophthalmitis in the early postoperative period, especially in cases with a previous history of herpetic eye disease. Administering full therapeutic doses of antiviral medication during the immediate postoperative period may potentially serve as a preventive measure to reduce the likelihood of such reactivation.
